# Exploring of miR-155-5p, miR-181b-5p, and miR-454-3p Expressions in Circulating Cell-Free RNA: Insights from Peripheral Blood of Uveal Malignant Melanoma Patients

**DOI:** 10.1007/s10528-024-10849-8

**Published:** 2024-06-24

**Authors:** Hassani Masoumeh, Doğan Tunay, Ödemiş Akdeniz Demet, Tuncer Samuray, Yazıcı Hülya

**Affiliations:** 1 Cancer Genetics Division, Oncology Institute, İstanbul University, Çapa-Fatih, 34093 Istanbul, Türkiye; 2 Department of Medical Pathology, Faculty of Medicine, Istinye University, Cevizlibağ-Zeytinburnu, 34010 Istanbul, Türkiye; 3 Department of Eye Diseases, Faculty of Medicine, İstanbul University, Çapa-Fatih, 34093 Istanbul, Türkiye; 4 Health Institutes of Türkiye, Türkiye Cancer Institute, Kadıköy, 34734 Istanbul, Türkiye; 5 Department of Medical Biology and Genetics, Faculty of Medicine, İstanbul Arel University, Merkez Efendi Mah, Eski Londra Asfalti.Cd., No 1/3, Cevizlibag, Zeytinburnu, 34010 Istanbul, Türkiye

**Keywords:** Uveal Melanoma, miR-155-5p, miR-181b-5p, miR-454-3p, Circulating cell free miRNA, Plasma

## Abstract

The identification of novel non-invasive biomarkers is imperative for the early diagnosis and monitoring of malignant melanoma. The objective of this study is to examine the expression levels of miR-155-5p, miR-181b-5p, and miR-454-3p in circulating cell-free RNA obtained from plasma samples of the 72 uveal malignant melanoma patients and to compare these levels with those of 72 healthy controls. The analysis showed that the expression level of the miR-181b-5p has increased 9.25 fold, and expression level of miR-155-5p has increased 6.67 fold, and miR-454-3p expression level has increased 4.14 fold in the patient group compared with the levels in the healthy control group (p = 0.005). It was found that the high expression levels of the three miRNAs were statistically significant in patients compared with in the healthy control group. The statistical evaluations between miRNA expression levels and clinical data showed that miR-155-5p had significant association with radiation therapy (p = 0.040), and miR-454-3p showed a significant association with smoking and alcohol use respectively (p = 0.009, and p = 0.026). The significantly elevated expression levels of miR-181b-5p, miR-155-5p, and miR-454-3p in the circulating cell-free RNA of plasma from uveal melanoma patients, in comparison to those in the healthy control group, suggest the potential usefulness of these biomarkers for both early diagnosis and disease monitoring. However, more extensive and future studies are needed to use these molecules in early diagnosis and disease monitoring.

## Introduction

Uveal melanoma (UM) is a type of intraocular cancer of adults and seriously impairs patients’ eyesight and the quality of life (Krantz et al. 2017). Approximately 2000 new uveal malignant melanoma cases are diagnosed annually in the United States of America (Materin et al. 2011). The tumor with a poor prognosis metastasizes in the early stages of the disease and shortens the survival time of patients (Shields et al. 2013; Shields and Shields 2015). Uveal melanoma spreads in the iris, ciliary bodies, and highly in the choroid region in the eye. Some studies indicated that there are some very rare melanoma tumors that are located in the conjunctiva region (Koc and Kiratli 2020; Wong et al. 2014). Such type of melanoma is classified as ocular melanoma (Seregard 1998; Brownstein 2004; Eskelin et al. 2000). Different methods are used in the treatment of uveal melanoma, including radiotherapy, Laser Therapy, and Excision, depending on the patient’s tumor area and size (Dogrusoz et al. 2017).

Although the diagnosis of uveal melanoma is possible during a clinical examination, the tumor is overlooked in many patients and the tumor may be found at an advanced stage (McLaughlin et al. 2005). Apart from its anatomical location, the higher mobility ability of the ciliary bodies and the excess number of vessels in this area not only complicate the early diagnosis, but also leads to the formation of an extravascular matrix pattern with a poor prognosis, and accordingly to the development of a highly metastatic potential (Chang et al. 1998; Johansson et al. 2010; Yan et al. 2009; Dithmar et al. 2000). This cancer with high tendency to metastasize, makes distant liver metastasis by 90–95% through hematogenous spread (Chen et al. 2011; Singh et al. 2001). 40–50% of melanoma patients die within several months due to metastasis owing to the way it spreads and its metastatic properties (Kujala et al. 2003). Therefore, there is a need for new non-invasive and highly sensitive biological biomarkers that can provide data with minimally invasive methods in uveal melanoma for the early diagnosis, prognosis, and monitoring of the disease, and also the development of new treatment protocols.

Various biological materials exhibiting the characteristics of liquid biopsy are present in different structures in our bodies. One of these biological materials is peripheral blood. Other body regions containing materials with liquid biopsy features include cerebrospinal fluid, saliva, pleural effusion, peritoneal ascitic fluid, urine, feces, tear fluid, breast milk, seminal fluid, vaginal and cervical discharge, and cooled and condensed exhaled breath (EBC). In the plasma or serum phase of peripheral blood, circulating cell free RNA molecules (cfRNA) [miRNA(microRNAs) and lncRNA(Long noncoding RNA), mRNA(Messenger RNA)], circulating cell-free DNA(cfDNA) molecules, i.e., fragmented nucleic acid molecules, exosomal vesicules (EV), proteins, TEP (Tumor Educated Blood Platelets), In the cellular phase of peripheral blood which is the lower phase, there are two subtypes of cell fractions. the circulating tumor cells (CTC) along with Neutrophil-engaged CTCs are found in the tumor cell fraction. In the subfraction outside the tumor cell, immune cells, CEC (Circulating Endothelial Cells), and CA fibroblasts (Cancer-Associated Fibroblasts) are present.

Currently, the most widely used materials for liquid biopsy include cell-free circulating nucleic acids (cfDNA/cfRNA), and circulating tumor cells (CTC) with extracellular vesicles (EV). Particularly, CTCs, EV, cfDNA, and cfRNA (miRNA) play a significant role in early diagnosis and disease monitoring. The major challenge in cancer is late diagnosis and the development of metastasis. Traditional tumor biopsies are only present at the time of diagnosis and are unfortunately not useful biological materials for monitoring and tracking the disease since they completely disappear after the first few treatments. Especially, liquid biopsy materials such as serum/plasma and saliva are commonly used biological materials with liquid biopsy properties. These materials are frequently employed as non-invasive biological markers in the early diagnosis of the disease, monitoring of progression, early detection of recurrences, determination of treatment response and metastatic risk, evolution of tumor tissue, and monitoring of minimal residual disease (MRD) (Eskelin et al. 2000; Jin and Burnier 2021; Carvajal et al. 2014; Luke et al. 2013).

It is important to investigate the miRNAs that will guide from a diagnostic and prognostic point of view in serum and plasma samples of patients for determining new biological markers in order to decrease the false negative and false positive clinical results in the diagnosis of uveal melanoma. The investigation of the circulating miRNAs is important for the early diagnosis as well as for monitoring each stage of the disease. Other than having non-invasive and easily accessible circulating biomarkers, their availability for investigation in any requested time interval with high precision and originality make these molecules worth examination (Chen et al. 2008; Gilad et al. 2008; Mitchell et al. 2008). MicroRNAs (miRNAs) are diminutive, non-coding molecules typically composed of 22–24 nucleotides. They serve as regulators of gene expression, modulating the suppression of gene expression and governing the post-transcriptional activity of target mRNAs (Vannini et al. 2018). These molecules exhibit both oncogenic and tumor suppressor properties and play a crucial role in the intricate process of cancer development (Bignotti et al. 2016; Jones-Rhoades and Bartel 2004). miRNAs whose expression is upregulated in tumors, also known as “oncomirs”, promote cancer progression by inhibiting the expression of tumor suppressor genes involved in different biological processes. Since miRNAs are highly stable molecules in different biological fluids such as serum/plasma, they can be used as a potential circulating biomarker (Gattuso et al. 2022). Currently, miRNA molecules are known to be used as the target molecule in cancer treatment in various cancer types, and as a biological biomarker in cancer diagnosis and monitoring of the treatment, and/or are known as the candidates for these procedures (Yao et al. 2019; He et al. 2020; Ward et al. 2013; Zhang et al. 2019).

As with many cancers, the development of uveal malignant melanoma involves the exposure of oncogenes and tumor suppressor genes to multi-stage genetic and epigenetic changes (Jager et al. 2020). Some studies reported that the small noncoding RNAs have a role in the development of uveal melanoma (Li et al. 2020). The expression levels of different miRNAs were found to have increased compared with the normal cells/normal tissues in malignant melanoma cell lines and tumor tissues in studies which used different methods such as microarray and NGS (Zhang et al. 2018; Peng et al. 2017; Li et al. 2015; Souri et al. 2021). Currently, miRNA molecules are known to be used as the target molecule in cancer treatment in various cancer types, and as a biological biomarker in cancer diagnosis and monitoring of the treatment, and/or are known as the candidates for these procedures.

The miRNAs named miR-181b-5p, miR-155-5p and miR-454-3p were reported to have high level of expression in uveal malignant melanoma cell lines (Zhang et al. 2018; Peng et al. 2017). These three miRNAs are oncogenic miRNAs and the target genes they affect *WEE1, ATM, PTEN* tumor suppressor genes, respectively. The molecules belonging to the miR-181 family are suggested to negatively control the cell cycle therefore they may be the drug targets (Zhang et al. 2018; Frixa et al. 2015; Wu et al. 2014). miR-155-5p has been shown to be overexpressed in malignant cell line strains in the uveal malignant melanoma cell line studies investigating the miR-155-5p, and suggested that miR-155-5p could have a role in proliferation, invasion and metastasis, and could be a therapeutic target (Peng et al. 2017).

The aforementioned studies showed that the miR-155-5p and miR-181b-5p, had overexpression only in uveal melanoma cell strains, however miR-454-3p was investigated in a very limited number of human tumor tissue and normal tissues in addition to uveal malignant melanoma cell strains (Zhang et al. 2018; Peng et al. 2017). There is no study in the literature which had identified the prognostic importance of miRNAs which were investigated in the plasma samples in the peripheral blood circulation of uveal malignant melanoma patients. In the present study, the expression levels of miR-155-5p, miR-181b-5p, and miR-454-3p were compared in plasma samples between 72 uveal malignant melanoma patients, and in an equal number of healthy controls, and the diagnostic and prognostic significance of these miRNAs was evaluated.

## Materials and Methods

### Study Populations

The present study was initiated after the approval of the Clinical Research Ethics Board (Dated 20.09.2019/ No: 16) of Istanbul University, Istanbul Faculty of Medicine. The study was performed on the plasma samples which were separated from the peripheral blood samples of uveal melanoma patients who presented to Istanbul University Oncology Institute, Department of Basic Oncology, and to the Department of Eye Diseases in Istanbul Faculty of Medicine. The experimental group consisted of 72 uveal malignant melanoma patients who presented to the Eye Diseases polyclinic in Istanbul University, Istanbul Faculty of Medicine in 2019, and the control group consisted of 72 healthy individuals who were matched for age, sex, and ethnicity with the patient group and had no history of cancer in the family in the last three generations. 3 out of 72 patients in the patient group were newly diagnosed to have the disease, the remaining patients were found in different stages of different treatment and follow up. All individuals included in the study were informed about the study before the peripheral blood samples were drawn, and their informed consent forms were received. The mean age of the patients was 54.68 years (± 13.69); the mean age of the healthy control group was 54.25 years (± 13.24), and the mean age of the patients at diagnosis was 48.19 years (± 12.97). There is no statistical difference between the mean age both in the control group consisting of healthy people and the group of patients with uveal malignant melanoma, and the groups showed similar distribution for age. The demographic, and clinical characteristics of the patients and the control group are given in Table 1.

**Table 1:** The demographic, and clinical characteristics of the patients

Patients	Characteristics	Number of Patients and Percentage, n (%)
Age(years)	≤ 60	26 (36.11%)
	> 60	46 (63.89%)
Histological Diagnosis	Iris Melanoma	7 (9.72%)
	Ciliary Bodies Melanoma	1 (1.39%)
	Choroidal Melanoma	56 (77.78%)
	Conjunctival Melanoma	3 (4.17%)
	Iris + Ciliary Bodies Melanoma	1 (1.38%)
	Choroidal + Ciliary Bodies Melanoma	4 (5.56%)
Stage	Early Stage (I, II)	38 (52.77%)
	Advanced Stage (III, IV)	34 (47.23%)
Metastasis (Liver, Kidney, Adrenal Gland)	Yes	4 (5.56%)
	No	68 (94.44%)
Risky Occupation	Yes	31 (43.06%)
	No	41 (56.94%)
Secondary Primary Tumors (Brain, Breast, Colon)	Yes	4 (5.56%)
	No	68 (94.44%)
Ciliary Bodies Involvement	Yes	6 (8.33%)
	No	66 (91.67%)
Smoking and Alcohol Intake	Yes	36 (50.00%)
	No	36 (50.00%)

### Cell Free RNA (ctRNA) Isolation from Plasma

First, the plasma was separated from the peripheral blood sample taken from the patient and healthy control groups. Plasma samples were stored in a liquid nitrogen tank for long-term storage. The plasma samples were removed from the nitrogen tank after adequate number of samples were obtained. The ctRNA extraction from plasma samples was performed using the Quick-cfRNA Serum and Plasma Kit (ZYMO RESEARCH) in accordance with the kit protocol. 1 mL Digestion Buffer and 50 µL Proteinase K were added onto 1 mL plasma extracted from the nitrogen tank and were incubated at 37 °C for 2 h. After incubation, 400 µL of Quick cfRNA binding buffer was added. 100% Propanol approximately 1.5 fold of the total volume in the tube was added, and loaded into the Spin-away column. The Spin-Away column was connected to the vacuum device, liquid contents were transferred to the column. Then, 600 µL of RNA Prep Buffer was added to the Spin-away column and washed, respectively. Then, 200 µL RNA Recovery Buffer was added to the column and the bottom liquid was discarded. The column was washed with 300 µL of 100% ethanol, 200 µL of RNA Prep Buffer, 200 µL and 400 µL RNA Wash Buffer twice. 15 µL DNase/RNase free dHO_2_ was added and centrifuged for 2 min at 12.000 g. The ctRNA was collected and immediately stored at − 70 °C.

### Measurement of the RNA Concentrations

The presence of the isolated total RNA was determined using the agarose gel electrophoresis. 5 µL sample of the obtained ctRNAs was mixed with 1 µL of 10X BPB (bromine phenol blue) and were loaded onto the gel and conducted at 150 Volts and imaged under UV. The concentration measurements of ctRNA were performed on a NanoDrop 2000 Spectrophotometer (THERMO SCIENTIFIC) device. The purity and concentration of the isolated RNA were determined by the absorbance at wavelengths of A260/A280 nm. The ctRNAs with an absorbance range of 1.5–2.0 OD were considered as pure/suitable ctRNA samples.

### cDNA Synthesis and Real-Time PCR

The cDNA synthesis from ctRNA using miRNA-specific Reverse Transcriptase enzyme with the help of ID3EAL cDNA Synthesis System (MIRXES) kit for Real-Time PCR reaction was carried out on the BioRad PCR device. Then, the obtained cDNAs were diluted using distilled water in a ratio of 1:10. The Real-Time PCR Reaction was performed on the MIC-Real Time PCR Device (MIC qPCR Cycler) using the diluted cDNAs belonging to the miR-U6 reference gene and with the target miRNAs of miR-155-5p, miR-181b-5p, and miR-454-3p. The expression level of each sample was calculated using the Ct (Threshold Cycle) value. The gene expressions were evaluated using the formula 2^−ΔΔCT^. The triplicate results for each sample were determined according to the average of the Ct values with this method. In accordance with the Formula 2^−ΔΔCT^, miR-U6 gene expression was taken as the reference, and the rate of increase or decrease of the expression levels of miR-155-5p, miR-181b-5p, and miR-454-3p was identified by comparing the rates for the patient group as in the control group.

### Statistical Analysis

The assumption of normality between the groups was evaluated using the Kolmogorov–Smirnov, and Shapiro Wilk tests in accordance with the statistical analyses using the Statistical Package for the Social Sciences (SPSS) v21.0 program. The p value was found as p < 0.05 in accordance with the normality assumption results, therefore the groups were suggested to have no normal distribution, and the use of the nonparametric Mann–Whitney U test was found applicable in the statistical analysis of all data.

## Results

The cell free RNAs extracted from plasma samples separated from the peripheral blood of 72 healthy individuals who were matched with the patient group in terms of age, sex and ethnicity were used as the control group with the samples of 72 patients with uveal malignant melanoma in our study. The results were evaluated among 72 patients with uveal malignant melanoma, and 72 healthy controls.

63.89% of the patient group was aged over 60 years, and 36.11% were aged below 60 years. The mean age of the patients was 54.68 years (± 13.69), and the mean age of the healthy control group was 54.25 years (± 13.24). The mean age at diagnosis in the patient group was calculated as 48.19 years (± 12.97). 77.7% of the patients were histologically classified as choroidal melanoma. 52.77% were in Stage I-II, and 47.23% were in Stage III-IV. Metastasis was present in 5.56% (4/72) of the patients at the time of diagnosis. 50% (2/4) of the patients with metastases had only liver metastases, 25% (1/4) had liver + kidney metastases, and 25% (1/4) had liver + kidney + adrenal gland metastases. In addition to uveal malignant melanoma, 5.56% (4/72) of the patients were diagnosed with secondary primary tumors, including breast cancer in 2, colon carcinoma in 1, and brain tumor in 1 individual. The ciliary body involvement was found in 97.97% of the patients.

**Fig. 1:**
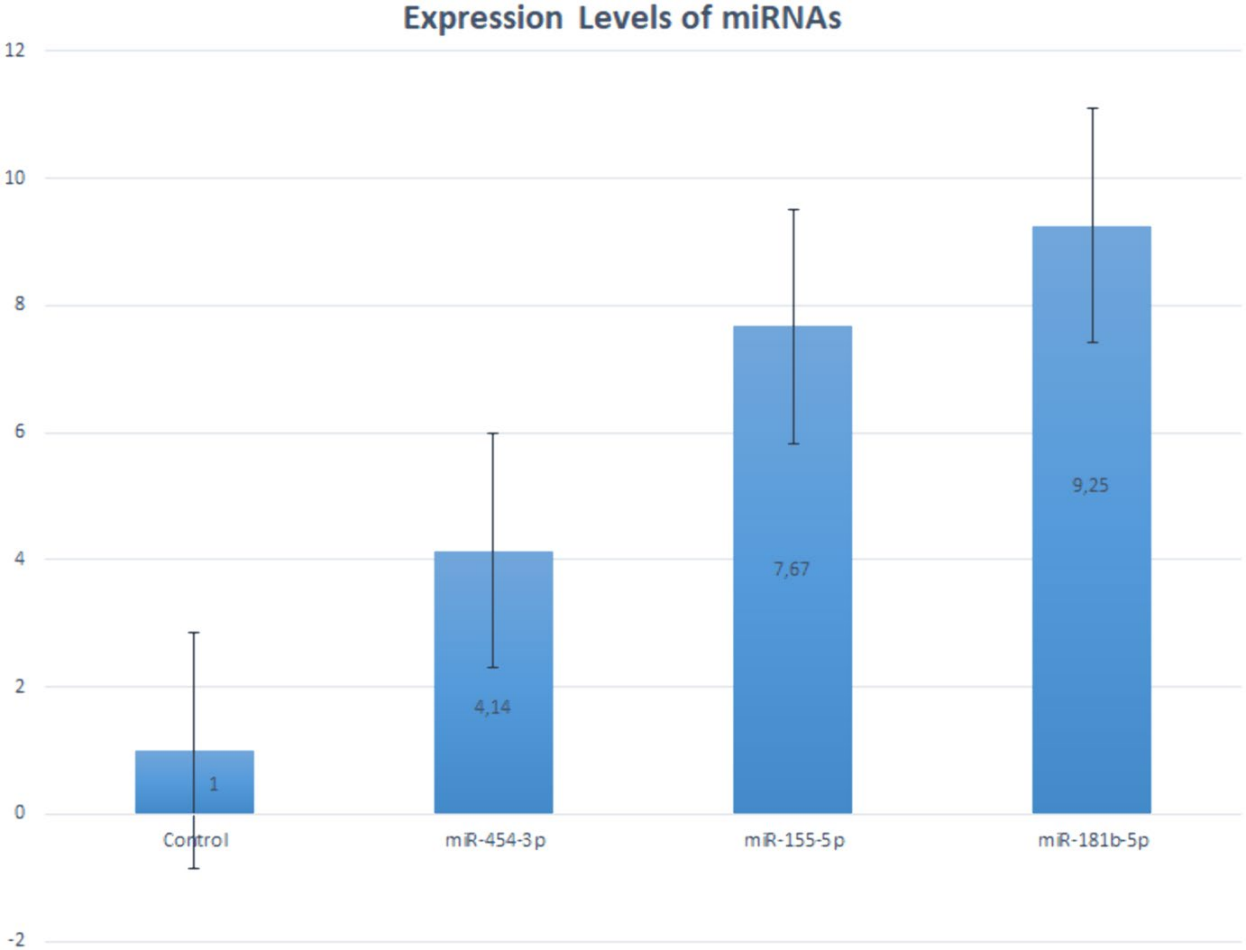
The comparison of the expression levels of miR-181b-5p, miR-155-5p and miR-454-3p in uveal melanoma patients with the healthy control group

The graphs indicating the increase or decrease in the expression levels (2-∆∆C t) in uveal malignant melanoma patients compared to the levels in the healthy group are given in Fig. 1. Considering the Fold & Change (|FC|≥ 2) value in the comparison of the gene expression levels of the uveal malignant melanoma patients with the results of the healthy control group showed that miR-181b-5p increased 9.25 fold, miR-155-5p by 7.67 fold and miR-454-3p increased 4.14 fold.

We found that the expression levels of miR-181b-5p, miR-155-5p, and miR-454-3p showed statistical difference (p < 0.05) in the patient group compared with the levels in the control group in the calculations based on the 2^−∆∆Ct^ values between the patient and control groups in accordance with the evaluations with the Mann–Whitney U test. The expression levels of miR-181b-5p, miR-155-5p and miR-454-3p in the uveal malignant melanoma patients were found statistically highly significant compared with the levels in the healthy controls (p = 0.005).

The association of the expression levels of miR-155-5p, miR-181b-5p, and miR-454-3p in patients with uveal malignant melanoma with the variables as the age, age at diagnosis, clinical stage, tumor size, metastasis status, treatment options as surgery, radiotherapy or chemotherapy, the risky occupational group status, and smoking and alcohol use were analyzed and evaluated using the Mann–Whitney U test. No significant association was detected with other variables except radiotherapy and smoking and alcohol use, with the expression levels of miR-155-5p, miR-181b-5p, and miR-454-3p, however, miR-155-5p expression level was found higher in patients receiving radiotherapy compared with the non-receiving patients, and compared with the healthy group, and this association was found statistically significant (p = 0.040). Furthermore, a statistical significance was found between the miR-454-3p expression level with smoking and alcohol use as P = 0.009, and P = 0.026, respectively (Tables 2, 3 and 4).

**Table 2:** The relationship between miR-155-5p expression levels and radiotherapy

miRNA-155-5p Expression Levels					
Expressions	Decreased n (%)		Increased n (%)	Total n(%)	
Radyotherapy					
Not received	1 (1.4%)		36 (50.0%)	37 (51.4%)	
Received	6 (8.3%)		29 (40.3%)	35 (48.6%)	
Total n(%)	7 (9.7%)		65 (90.3%)	72 (100%)	p = 0.040

**Table 3:** The relationship between miR-454-3p expression levels and smoking

miRNA-454-3p Expression Levels					
Expressions	Decreased n (%)		Increased n (%)	Total n(%)	
Smoking					
Yes	2 (2.8%)		33 (45.8%)	35 (48.6%)	
No	11 (15.3%)		26 (36.1%)	37 (51.4%)	
Total n(%)	13 (18.1%)		59 (81.9%)	72 (100%)	p = 0.009

**Table 4:** The relationship between miR-454-3p expression levels and alcohol intake

miRNA-454-3p Expression Levels				
Expressions	Decreased n (%)	Increased n (%)	Total n(%)	
Alcohol intake				
Yes	11 (15.3%)	58 (80.6%)	69 (95.8%)	
No	2 (2.8%)	1 (1.4%)	3 (4.2%)	
Total n(%)	13 (18.1%)	59 (81.9%)	72 (100%)	p = 0.026

It was striking to find out that the expression levels of miR-155-5p, miR-181b-5p, and miR-454-3p in patients with metastases at the time of diagnosis and in patients with secondary primary tumors were at higher extremes than the mean levels in the healthy control group. In addition, the highest expression level of these miRNAs was observed in 3 patients who had just been diagnosed and had not yet undergone any treatment or surgical procedure. We suggest that these extremely higher levels of miR-155-5p, miR-181b-5p, and miR-454-3p expression are formed by the effect of the metastatic cells in the circulation in addition to the role of the active uveal melanoma tumor cells, and confirms that miRNAs have diagnostic power in each stage of the disease.

### String Analysis

**Fig. 2:**
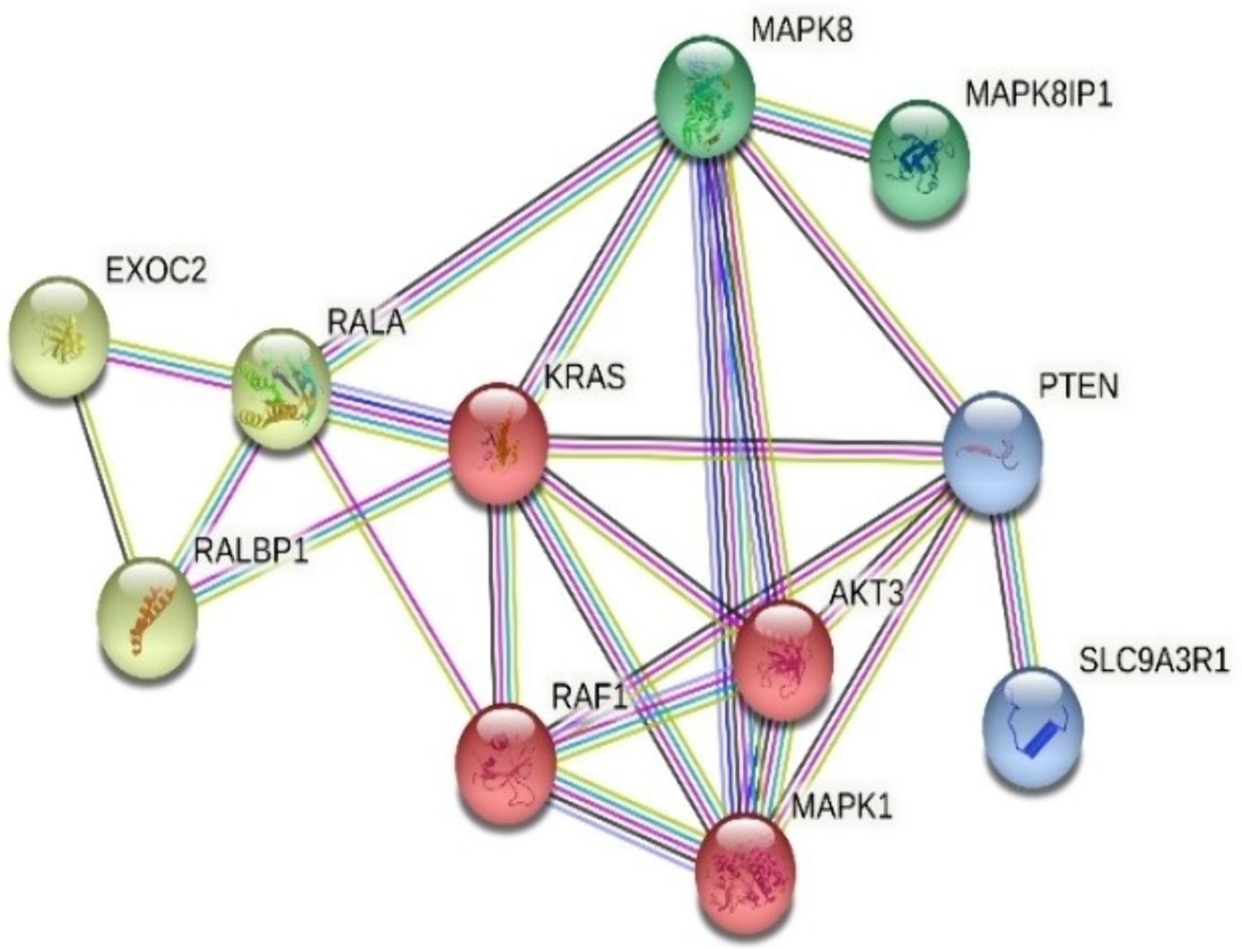
STRING analysis for target genes of miR-181b-5p (p value: 0.000118)

The miR-155-5p, miR-181b-5p, and miR-454-3p and their target genes investigated in the present study were determined using the “mirTarBase” and “TargetScan” databases. The target genes of miR-155-5p were *FOS, ETS1, TP53INP1, MDM2, MYB, RAP1B, CBL* and target genes of miR-181b-5p were found as *RALA, PTEN, KRAS, MAPK1, MAPK8, AKT3*, and the target genes of miR-454-3p were *MDM4, PTEN, WNT1, WNT2B, MAPK1, MET,* and *HGF*. The STRING (Protein–protein interaction analysis) analysis was performed to determine the interaction between miR-155-5p, miR-181b-5p, and miR-454-3p and the targets of these genes. The STRING analysis results showed a strong interaction between the genes targeted by miR-181b-5p and the associated proteins with the KRAS and MAPK1 proteins in the RAF/MAPK pathway (p = 0.000118). In addition, a strong interaction was found between MAPK1, and MAPK8 in the same analysis. Furthermore, the strong and significant associations of KRAS and RALA; KRAS with MAPK1; MAPK1 with MAPK8; MAPK8 with AKT3 proteins were also evaluated as remarkable in this analysis (p < 0.05) (Fig. 2).

**Fig. 3:**
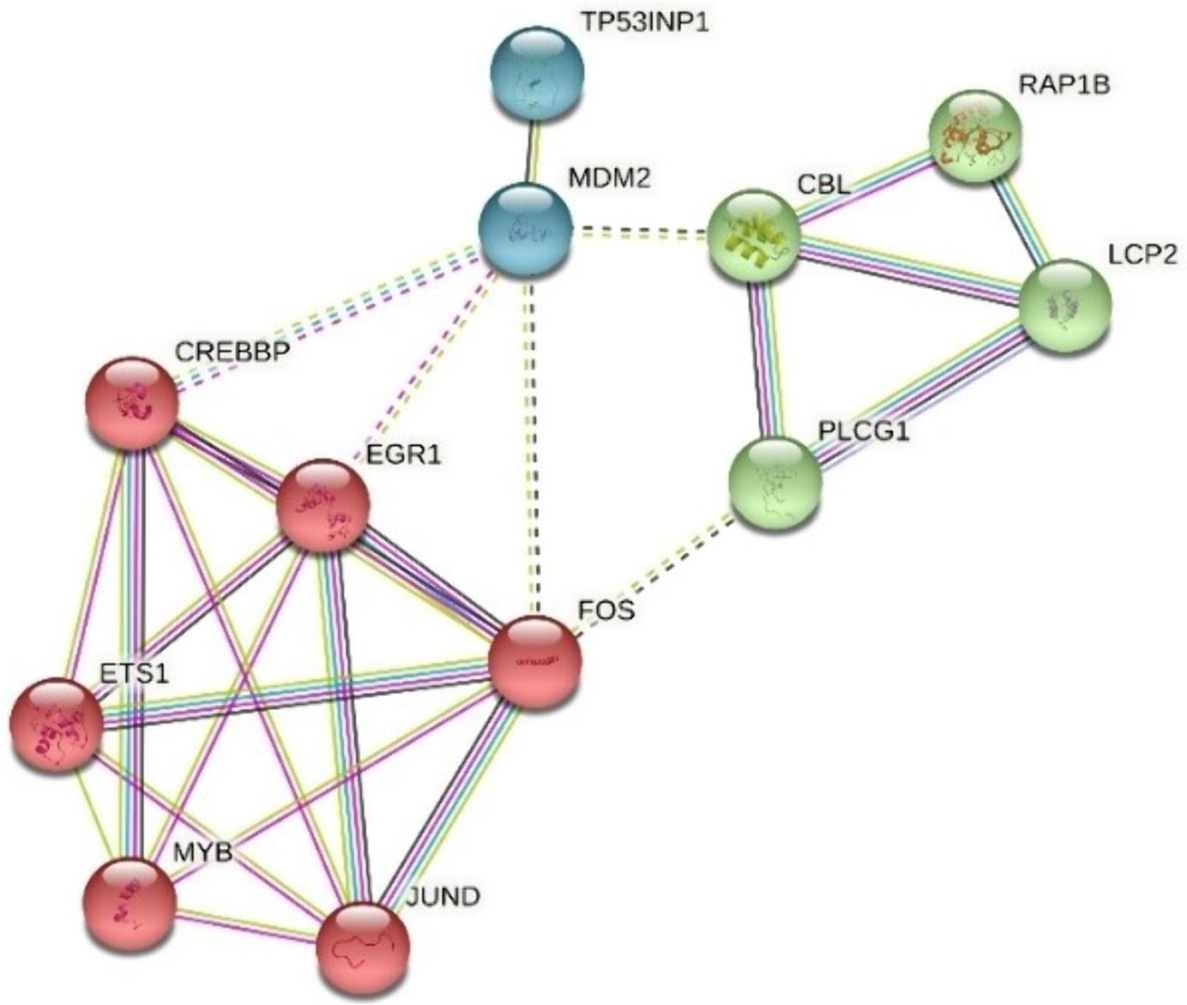
STRING analysis for target genes of miR-155-5p (p value: 0.000399)

The results of the STRING analysis performed for miR-155-5p showed that the molecules that interact most with miR-155-5p were JUND, FOS and ETS1 molecules. Although the FOS molecule intensively interacted with JUND, it also interacts with CBL, RAP1B, and LCP2 proteins via PLCG1 (p < 0.05) (Fig. 3). We found that the interaction of FOS with PLCG1 was also indirectly established via MDM2, which was remarkable.

**Fig. 4:**
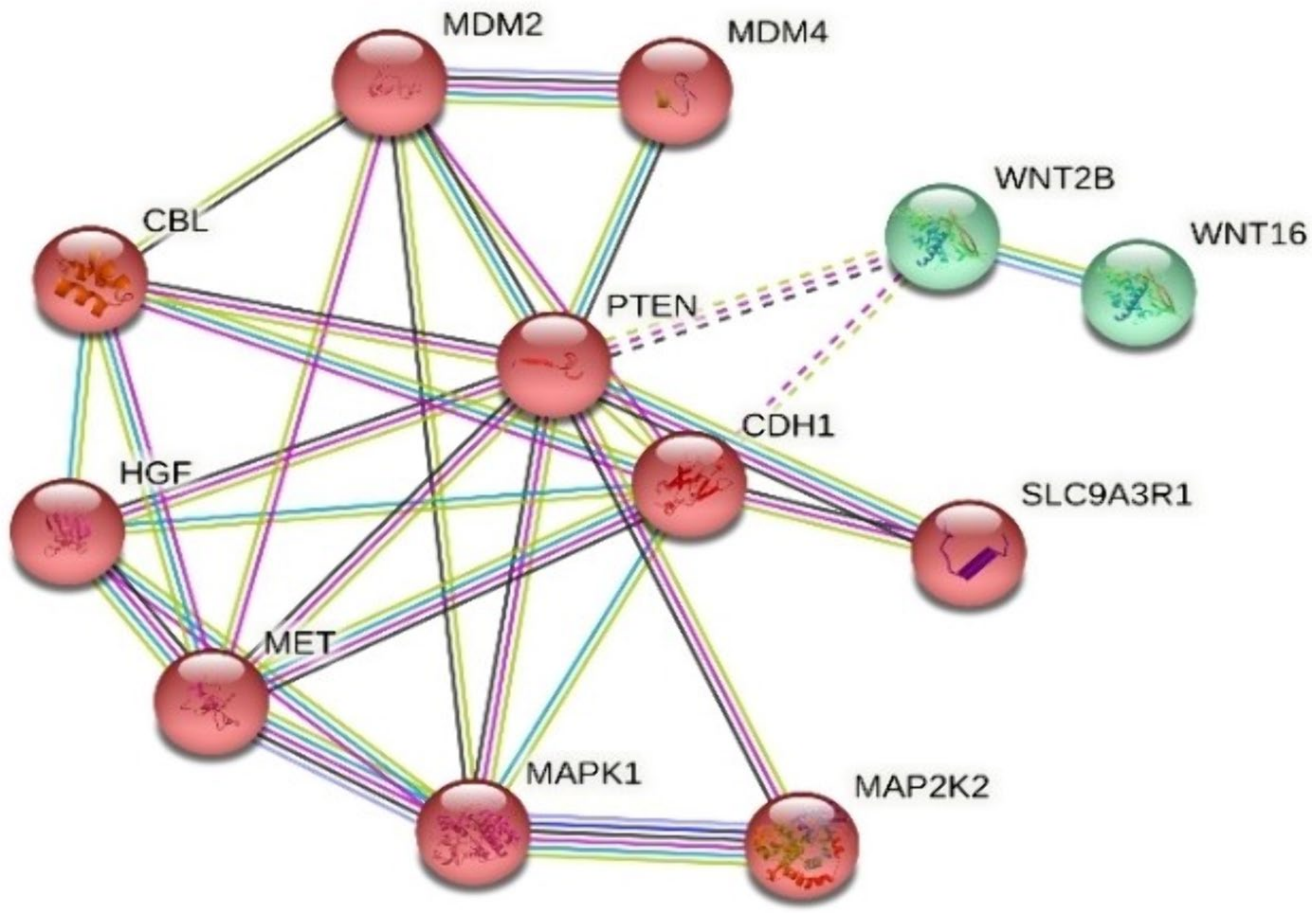
STRING analysis for target genes of miR-454-3p (p value: 0.000129)

The STRING analysis performed for the target proteins of miR-454-3p showed that the greatest interaction was between the MET and HGF; and there was a significant association between MET and CDH1; PTEN and MDM4; MDM4 and MDM2 molecules (p = 0.000129). The studies in the literature reported that there were interactions between the MDM2, FOXO and TP53 proteins in the PTEN and MDM4 signal transduction pathway. An intense association was detected between the MAPK1 and MAP2K2 molecules in the performed analysis. In addition, the analysis also showed that the WNT1 and WNT2B molecules, which have the closest sequence similarity, were also in close interaction (p < 0.05) (Fig. 4).

**Fig. 5:**
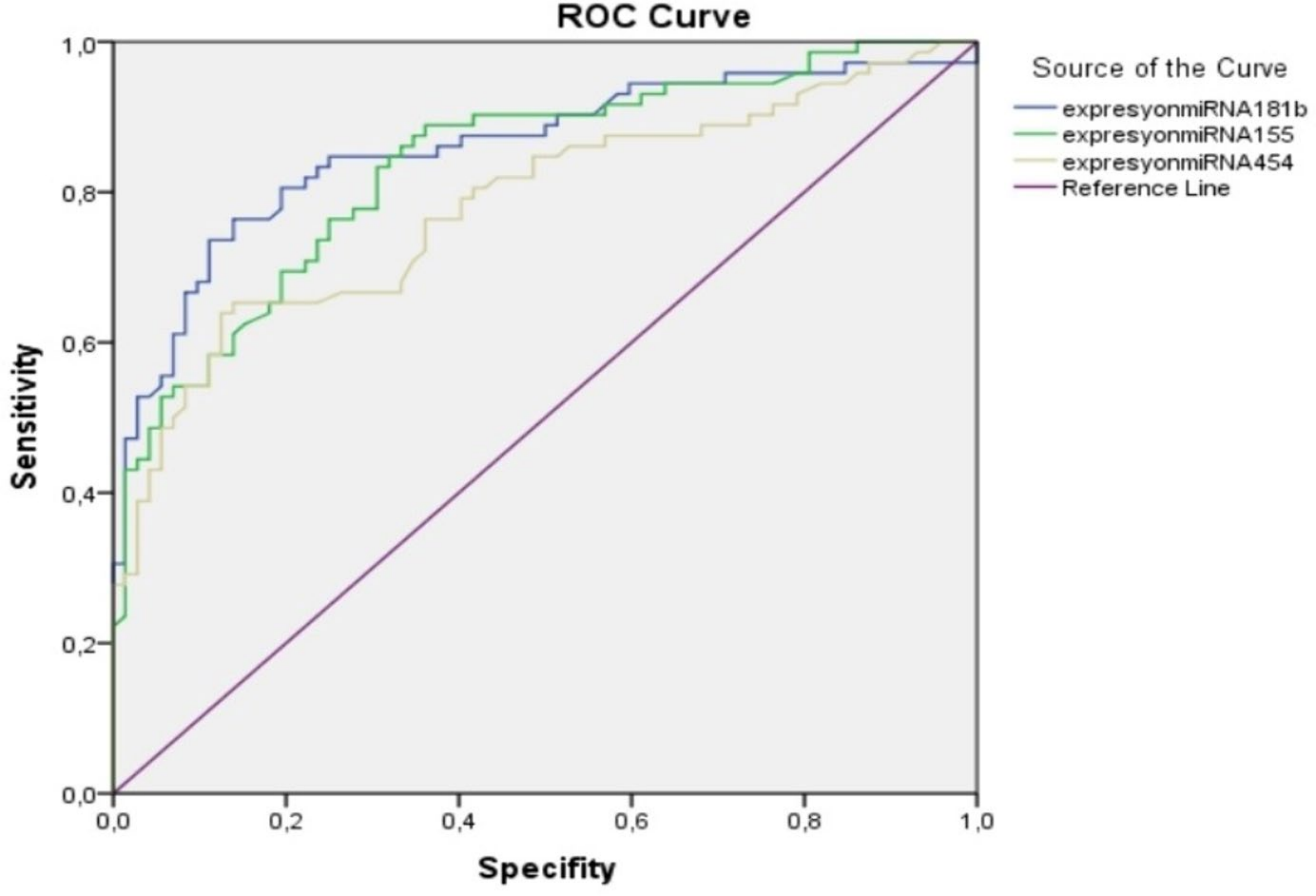
ROC analysis curve of miR-181b-5p, miR-155-5p and miR-454-3p

We examined the diagnostic performance of the miR-155-5p, miR-181b-5p, and miR-454-3p molecules as the candidate biomarkers -in other words, the differentiation power of the uveal malignant melanoma patients with the healthy control group using the Receiver Operator Characteristics (ROC) analysis. In Fig. 5, the ROC-AUC values determined for each miRNA, and 95% CI (confidence interval), and the diagnostic power of the molecules miR-155-5p, miR-181b-5p, and miR-454-3p in the diagnosis of patients with malignant uveal melanoma were found statistically highly significant (p = 0.000) (Fig. 5).

## Discussion

The expression data of human genes in the current studies show that miRNAs have an important role in the occurrence and progression of cancer (Zhang et al. 2007; Aughton et al. 2020; Catalanotto et al. 2016; He and Hannon 2004). Various studies have shown that the miRNAs are the effective molecules that can be used in the diagnosis and follow-up of cancer and in the treatment of cancer as a therapeutic agent or therapeutic target (Erson and Petty 2009; Sochor et al. 2014; Lagana et al. 2010; Lamy et al. 2006). To date, the miR-155-5p and miR-181b-5p in many cancer types were shown to have high expression in the serum samples of high-risk breast cancer patients before surgery and/or treatment, and different studies have shown various times that the expression levels of these miRNAs were decreased after treatment (Sochor et al. 2014) (Liu et al. 2013). Researchers in another study reported that the expression level of miR-155-5p in the circulating serum samples of patients with esophageal cancer increased compared with the levels in healthy individuals, and might be used as a non-invasive biologic biomarker (Liu et al. 2012). The studies associated with miR-454-3p expression reported that the miR-454-3p expression level showed differences in the cell lines of liver carcinoma (HCC) (Li et al. 2019a), cervical cancer (Song et al. 2019), breast cancer (Ren et al. 2019), glioma (Shao et al. 2015) and uveal melanoma in the literature. The abnormal miRNA expression levels shown in the uveal melanoma cell lines and tumor tissue were reported as closely associated with cell proliferation, invasion, and metastasis in different studies (Zhang et al. 2018; Peng et al. 2017; Eedunuri et al. 2015; Li et al. 2014; Li et al. 2019b; Sun et al. 2015; Venza et al. 2016; Wang et al. 2018a; Wang et al. 2018b; Wang et al. 2018c; Zhou et al. 2016; Ling et al. 2017). The investigation revealed a notable downregulation of miR-181b-5p in high-risk uveal melanoma cases. Furthermore, within the scope of the same study, a set of genes including *DERL1, FBXL17, MBOAT2,* *PLAG, SLC7A,* and *YTHDF3 *were identified as target genes susceptible to modulation by miR-181b-5p in tumor tissue of uveal melanoma patients (Smit et al. 2019). The bioinformatics and functional analysis results in a study which investigated the miR-181b-5p in uveal melanoma showed that miR-181b-5p precipitated the cell cycle by targeting the CTDSPL (small phosphatase-like molecule) of the carboxy-terminal region and created an oncogenic effect (Zhang et al. 2018). Researchers reported that the molecules of the miR-181 family, which are known to negatively control the cell cycle, may be the drug targets (Zhang et al. 2018). Similarly, miR-155-5p has been shown to create an oncogenic effect by promoting cell proliferation in uveal melanoma (Peng et al. 2017). Researchers in the studies investigating the miR-155-5p in uveal malignant melanoma cell lines suggested that miR-155-5p was overexpressed in tumor tissue and may be a therapeutic target (Peng et al. 2017). In many studies investigating the uveal malignant melanoma reported in the literature that different miRNAs act as tumor suppressors and/or oncogenes in uveal malignant melanoma. The miR-367, miR-21, miR-21a, miR-155-5p, miR-181b-5p, miR-92a-3p were shown to have oncogenic effect in uveal malignant melanoma cell lines and tumor tissue, however, miR-137, miR-144, miR145, miR-296-3p and miR-23A were shown to have tumor suppressor role (Li et al. 2020). Most studies summarized so far have been conducted on uveal malignant melanoma cell lines (Zhang et al. 2018; Peng et al. 2017; Eedunuri et al. 2015; Li et al. 2014; Li et al. 2019b; Sun et al. 2015; Venza et al. 2016; Wang et al. 2018a; Wang et al. 2018b; Wang et al. 2018c; Zhou et al. 2016; Ling et al. 2017), there was no study investigating the expression levels of miRNAs on ctRNA in plasma samples of patients in the literature.

We investigated the expression levels of miR-181b-5p, miR-155-5p and miR-454-3p on the ctRNAs differentiated from the plasma samples of the uveal malignant patients, and of the healthy controls who were matched with the patient group for age, sex, and ethnicity with no history of cancer in the past three generations in the family. We found that the miR-181b-5p was increased 9.25 fold, miR155 increased 7.67 fold, and the miR-454-3p was increased 4.14 fold in the comparison between the patient and healthy control groups who had no statistical difference between the mean age. The higher levels of miRNA expression, which were found statistically significant, could suggest that miRNAs have oncogenic properties considering the activities of the targeted genes. In our investigation, we identified a 9.25-fold increase in miR-181b-5p expression levels in plasma samples collected from uveal melanoma patients compared to those from healthy controls. Conversely, Smit et al., utilizing TCGA data and concentrating on tumor tissue samples from individuals with high-risk uveal melanoma, reported a downregulation in miR-181b-5p expression (Smit et al. 2019). These conflicting findings suggest that the disparate biological materials and population studied examined in both studies may account for the observed disparities.

We found no statistical significance between the expression levels of miR-181b-5p, miR-155-5p and miR-454-3p with having a risky occupation, tumor size, clinical stage, tumor location and metastasis status, however, there was a statistical significance between high miR-454-3p expression level with smoking and alcohol use, and between high miR-155-5p expression level with radiotherapy in our study. Demonstration of the higher expression levels of miR-181b-5p, miR-155-5p and miR-454-3p molecules on ctRNA, in all stages, and on the histological subtypes of uveal malignant melanoma suggest that these molecules have a diagnostic value in all stages, and histological subtypes. This result was confirmed by the Receiver Operator Characteristics (ROC) analysis performed in our study. The ROC analysis showed that the ability of the miR-181b-5p, miR-155-5p and miR-454-3p molecules to differentiate between the patient diagnosed with uveal melanoma and healthy control group was statistically significant (P = 0.000). All these results suggest that the higher expression levels of miR-181b-5p, miR-155-5p and miR-454-3p detected in plasma can be used as non-invasive biological biomarkers both in the diagnosis and follow-up of the disease. It will be meaningful to evaluate the power of these molecules, whose diagnostic power has been proven in our study, to be a prognostic and therapeutic target/agent by large-scale future studies.

We also identified the target genes and gene pathways of miR-181b-5p, miR-155-5p and miR-454-3p molecules which were found to have higher expression in ctRNAs of the plasma samples of uveal malignant melanoma using the STRING analysis. The KRAS and MAPK1 protein interactions were found strong between the genes targeted by the miR-181b-5p and between their associated proteins (p value: 0.000118). These two proteins are involved in the RAF/MAPK pathway (Chakraborty et al. 2016) and are among the most deteriorated pathways in cancer. We also found that there is a strong connection between the KRAS and RALA proteins in addition to MAPK1 and MAPK8 proteins of miR-181b-5p according to our STRING analysis. Furthermore, our STRING analysis revealed that miR-181b-5p influences distinct target genes in plasma compared to tumor tissue contexts, as indicated by the study conducted by Smit et al. (Smit et al. 2019).

The STRING analysis performed in plasma samples of uveal melanoma patients for miR-155-5p showed that the molecules with the most interaction of the miR-155-5p were CBL and RAP1B proteins along with FOS and ETS1 (p value: 0.000399). These proteins, which are effective in the MAPK and PI3K pathways, coordinate the normal functioning of that gene pathway. The increase in certain proteins due to the overexpression of miR155 is known to be effective in the development of cancer by working in the direction of proliferation of the gene pathway (Basuyaux et al. 1997). Our study results are totally compatible with this data.

The STRING analysis performed in plasma samples of uveal melanoma patients for miR-454-3p showed that the MET and HGF molecules were the target proteins that mostly interact with miR-454-3p (p value: 0.000129). Researchers in previous studies reported that MET and HGF molecules regulate various physiologic process including proliferation, morphogenesis, invasion, and survival with the receptor tyrosine kinase activity which transfer the signals from extracellular matrix to the cytoplasm through HGFR-HGF interaction (Danilkovitch-Miagkova and Zbar 2002; Gentile et al. 2008). The activation of PI3K-AKT-mTOR signaling pathways are known to be performed by the MAPK pathway with the auto-phosphorylation of the intracellular MET (Liu et al. 2020). These activated pathways play a role in the development of cancer by leading to cell survival by cell proliferation (Liu et al. 2020). The detection of higher level of miR-454-3p molecule in the peripheral blood of patients with uveal malignant melanoma supports that malignant formation occurs by this mechanism. The STRING analysis also showed that miR-454-3p interacts with MDM2, FOXO and TP53 proteins in the PTEN and MDM4 signaling pathways, as well as molecules in different pathways such as WNT1 and WNT2B. These results suggest that miR-454-3p is an extremely effective molecule that affects different gene pathways in carcinogenesis. All these protein–protein interactions should be individually investigated in future studies.

The weaknesses of our study were that the miR-155-5p, miR-181b-5p, and miR-454-3p molecules which were proven to be used for diagnostic purposes were not investigated in benign and other malignant eye tumors, and the peripheral blood samples taken from patients in specific periods were not investigated for identifying their prognostic value. However, the aim is to examine and evaluate these missing points in future studies.

In conclusion, our study is the first to investigate the expression levels of miR-155-5p, miR-181b-5p, and miR-454-3p in the ctRNA in the peripheral bloodstream of patients with uveal malignant melanoma. This study showed that the miR-155-5p, miR-181b-5p, and miR-454-3p molecules can distinguish uveal malignant melanoma patients from healthy people and are diagnostically valuable. The detectability of these molecules in plasma suggests that they can be used as the non-invasive biomarkers. The prognostic and therapeutic target/drug potential of these molecules, whose diagnostic power has been proven by our study, will be evaluated in future studies.

## Funding

This study was funded by the Scientific Research Projects Coordination Unit of Istanbul University (Grant Number: TDK-2019-35336).

## Data Availability

The datasets used and/or analyzed during the current study are available from the corresponding author on reasonable request.

## Competing Interests

The authors declare that they have no competing interests.

## Ethical Approval

All procedures performed in studies involving human participants were in accordance with the ethical standards of the institutional and/or national research committee and with the 1964 Helsinki declaration and its later amendments or comparable ethical standards.

## Patient Consent

Written informed consent was provided by the parents/guardians of the participants included in the study. The study was approved by the Ethics Committee of Istanbul Medical Faculty in Istanbul University (Approval document: Dated 20.09.2019/ No: 16).

## Consent to Participate

“Not applicable” In this study, no photographs of any organ or part of the body of the patients were used.
